# Optimization and Validation of a QuEChERS-Based Method Combined with Gas Chromatography–Tandem Mass Spectrometry for Analyzing Pesticide Residues in Edible Insect Samples

**DOI:** 10.3390/molecules30112293

**Published:** 2025-05-23

**Authors:** Phannika Tongchai, Nootchakarn Sawarng, Anurak Wongta, Udomsap Jaitham, Kunrunya Sutan, Saweang Kawichai, Chuleui Jung, Bajaree Chuttong, Surat Hongsibsong

**Affiliations:** 1 School of Health Sciences Research, Research Institute for Health Sciences, Chiang Mai University, Chiang Mai 50200, Thailand; phannika_tongchai@cmu.ac.th (P.T.); anurak.wongta@cmu.ac.th (A.W.); udomsap_j@cmu.ac.th (U.J.); 2 Environmental, Occupational Health Sciences and NCD Center of Excellence, Research Institute for Health Sciences, Chiang Mai University, Chiang Mai 50200, Thailand; kunrunya.s@cmu.ac.th (K.S.); sawaeng.k@gmail.com (S.K.); 3 Faculty of Public Health, Chiang Rai Rajabhat University, Chiang Rai 57100, Thailand; nootchakarn.saw@crru.ac.th; 4 Department of Plant Medicals, Gyeongkuk National University, Andong 36729, Republic of Korea; cjung@gknu.ac.kr; 5 Meliponini and Apini Research Laboratory, Department of Entomology and Plant Pathology, Faculty of Agriculture, Chiang Mai University, Chiang Mai 50200, Thailand

**Keywords:** pesticide residues, edible insects, QuEChERS, GC-MS/MS, food safety, method validation, matrix effects, extraction optimization

## Abstract

The increasing popularity of edible insects as a sustainable food source necessitates stringent safety measures to monitor pesticide contamination. This study aimed to assess and enhance a QuEChERS-based extraction method coupled with gas chromatography–tandem mass spectrometry (GC-MS/MS) for the quantification of pesticide residues in edible insects (bamboo caterpillars, house crickets, silkworm pupae, giant water bugs, and grasshoppers) by combining multiple individual insect specimens into a single, homogenized sample—five replicates were tested. The method was optimized by evaluating various extraction parameters and showed strong linearity for all 47 target pesticides, with correlation coefficients (R^2^) ranging from 0.9940 to 0.9999. The limits of detection (LODs) varied between 1 and 10 µg/kg, while the limits of quantification (LOQs) ranged from 10 to 15 µg/kg. Recovery studies conducted at three fortification levels (10, 100, and 500 µg/kg) revealed recoveries ranging from 64.54% to 122.12%, that over 97.87% of the pesticides exhibited satisfactory recoveries within the range of 70–120%, and relative standard deviations (RSDs) below 20%, between 1.86% and 6.02%. Matrix effects (%MEs) range from −33.01% to 24.04%, and to those that experienced no effect. More than 94% of the analytes showed minimal ion suppression or enhancement. These results conform to the SANTE guidelines for monitoring pesticide residues in edible insects, enhancing food safety standards and safeguarding consumer protection.

## 1. Introduction

The global shift towards sustainability and eco-friendly food options has generated increasing interest in edible insects as a viable alternative protein source [1]. These insects provide a rich source of high-quality protein and essential fatty acids, vitamins, and minerals. Their production is notably more resource-efficient than that of conventional livestock, requiring significantly less land, water, and feed, which contributes to a more sustainable food system [2]. In numerous regions, especially across Asia, Africa, and Latin America, consuming insects has a long-standing history. In Thailand, the consumption of insects is deeply ingrained in cultural and culinary practices. Commonly consumed species include house crickets (*Acheta domesticus*), grasshoppers (*Melanoplus foedus*), silkworm (*Bombyx mori*) pupae, bamboo caterpillars (*Omphisa fuscidentalis*), and diving beetle (*Cybister limbatus*) [3].

While edible insects offer potential benefits for food security and nutrition, they also pose food safety concerns due to the risk of pesticide contamination. Insects are exposed to pesticides both directly from treated areas and indirectly through their contaminated diet [4]. Furthermore, specimens gathered from their natural environments might be influenced by agricultural runoffs or the use of pesticides within their ecosystems [5]. Several studies have reported the presence of pesticide residues in edible insect samples, specifically in food products made from *A. domesticus* collected in Vietnam, with some residues belonging to pesticides that are either banned or restricted in the country [6]. Previous studies identified nine agrochemicals in six species of edible insects, including insecticides, herbicides, and fungicides, with some species exceeding maximum residue limits for certain chemicals. Notably, the Nigerian cricket (*Brachytrupes membranaceus*) contained detectable residues of several pesticides currently in use, although the levels of most residues remained below the established maximum residue limit of 0.01 mg/kg, indicating compliance with safety standards [7]. Moreover, a study analyzing various species of edible insects, including mealworms (*Tenebrio molitor*), crickets, silkworm pupae, and grasshoppers, found that some samples contained high microbial counts, particularly those that were raw or inadequately processed. Chemical analysis also revealed the presence of heavy metals, including lead, cadmium, and arsenic, although the levels remained within Canadian safety limits. Mycotoxins and pesticide residues were either undetectable or present at very low concentrations [8]. The study of pesticides in edible insects is complicated by the elevated levels of fat and protein in the matrix [9]. Chromatographic techniques for quantifying pesticide residue concentrations often necessitate comprehensive lipid removal before sample introduction into the system [10]. Consequently, the cleanup protocol must be refined to eliminate sample-derived matrices efficiently. Various extraction methods have been developed for this purpose, including solid-phase extraction (SPE), liquid–liquid extraction (LLE), solid-phase microextraction (SPME), and the QuEChERS method, which stands for quick, easy, cheap, effective, rugged, and safe [11].

The QuEChERS method is widely recognized for its versatility and effectiveness in extracting pesticide residues from various food matrices. Its advantages include high recovery rates, reduced solvent consumption, and suitability for high-throughput workflows. This method typically involves extraction with acetonitrile and a salt mixture to induce phase separation, followed by cleanup using dispersive solid-phase extraction (dSPE) with sorbents such as primary secondary amine (PSA) and anhydrous magnesium sulfate (MgSO_4_). Additional sorbents, such as C18 or graphitized carbon black (GCB), are sometimes employed to enhance cleanup, particularly for complex matrices [12,13,14,15]. Given the variability in fat content and matrix complexity among edible insect species, adapting and optimizing the QuEChERS protocol is essential to ensure accurate and reliable pesticide residue analysis.

Pesticide residue extraction methods are often analyzed using chromatographic techniques, such as gas chromatography–mass spectrometry (GC-MS/MS) or liquid chromatography–mass spectrometry (LC-MS/MS). Recently, liquid chromatography paired with quadrupole time-of-flight mass spectrometry (LC-QTOF/MS) has emerged as a powerful approach for both non-targeted and targeted pesticide screening in complex food matrices, owing to its enhanced resolution, accuracy, and sensitivity [16]. Studies have demonstrated the successful application of QuEChERS, combined with LC-MS/MS or GC-MS/MS, to detect pesticides in high-fat or complex matrices, such as rice, vegetable oils, and mealworms [17,18]. Previous studies using liquid chromatography–tandem mass spectrometry (LC-MS/MS) were developed to analyze 353 pesticides in *Tenebrio molitor* larvae. The method achieved limits of quantitation ≤10 μg/kg, with over 90% recovery and negligible matrix effects for most pesticide analyses [19].

The purpose of this study is to develop and validate an optimized QuEChERS-based extraction method combined with GC-MS/MS for multiresidue pesticide analysis in edible insects. The method was applied to real insect samples to assess potential pesticide contamination, supporting food safety monitoring efforts for insects, which are increasingly recognized as sustainable future food sources.

## 2. Results and Discussion

### 2.1. Optimization of Sample Extraction Procedure

The extraction of pesticide residues from edible insect matrices presents a significant analytical challenge due to their high protein and lipid content. In particular, lipids are highly soluble in organic solvents, which facilitates their co-extraction. To ensure accurate analysis, it is essential to purify the sample using appropriate pretreatment procedures prior to gas chromatography (GC) analysis [17,19]. The QuEChERS method was modified to use the optimal solvent/sample ratio. A standard mixed solution of 47 selected pesticides (100 μg/mL) was spiked into edible insect samples (2.5, 5.0, and 10.0 g). Each sample was transferred to a 50 mL centrifuge tube, to which varying amounts of acetonitrile (ACN) (5.0, 10.0, and 15.0 mL) were added, followed by 5 mL of water. The mixtures were then agitated for 5 min. Subsequently, a QuEChERS extraction package comprising 6 g of magnesium sulfate (MgSO_4_) and 1.5 g of sodium citrate (Na_3_C_6_H_5_O_7_) was incorporated to enhance phase separation and optimize cleanup efficacy.

**Figure 1:**
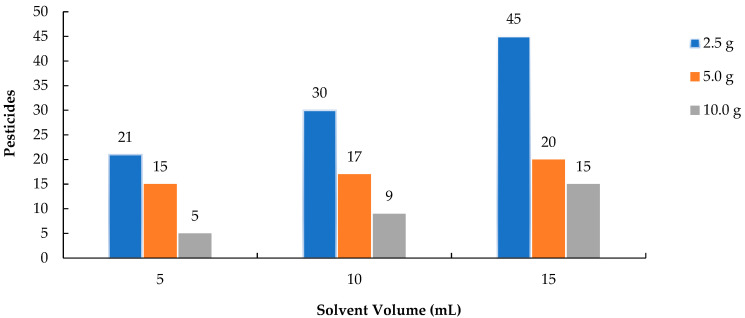
Number of pesticides for extractions with different amounts analyzed by GC-MS/MS.

The number of pesticides extracted increased significantly with the addition of higher volumes of ACN across all sample sizes, as illustrated in Figure 1. In the 2.5 g sample, the number of detectable pesticides increased markedly from 21 (extracted with 5 mL of ACN) to 45 (with 15 mL of ACN), indicating that extraction efficiency improves with increased solvent volume. The 5.0 g and 10.0 g samples exhibited a similar trend, though their overall recovery rates were lower. Using a larger volume of solvent relative to the sample size is especially critical for smaller samples, as it facilitates the separation of lipophilic pesticide residues into the extraction solvent [20,21,22]. This study demonstrated that increasing the volume of ACN significantly enhanced the extraction efficiency of lipophilic pesticides. Similar to the findings of Shin et al. (2020) [19], who demonstrated that a solvent-to-sample ratio of 3:1 or greater substantially improved the recovery of lipophilic pesticides in mealworms, this study found that increasing the volume of acetonitrile led to a marked increase in the number of detectable analytes, especially in small sample sizes.

The extraction of lipophilic (fat-loving) pesticides was effectively enhanced by using a greater volume of acetonitrile (ACN) in this study. This observation aligns with the findings of Lee et al. [23], who demonstrated that using a higher solvent-to-sample ratio facilitates more efficient migration of pesticides from complex insect matrices into the organic layer. A larger volume of ACN promotes efficient partitioning, thereby improving analyte transfer, as the adipose layer in the insects acts as a reservoir for lipophilic compounds.

This study utilized freeze-drying (lyophilization) as a sample preparation technique to enhance analytical precision and maintain the integrity of pesticide residues in edible insect matrices. Freeze-drying efficiently eliminates water content without applying heat, thus reducing the likelihood of thermal degradation or enzymatic modification of target analytes, which is particularly crucial for thermolabile or pH-sensitive pesticides [20]. Moreover, dry samples offer enhanced control over sample weight and solvent ratios, which is crucial for method optimization. Utilizing smaller sample sizes (e.g., 2.5–5.0 g) is especially beneficial for insects, which frequently produce less biomass and possess a higher fat content that may hinder extraction efficiency [19]. Our study confirms that lyophilized insect samples, when adequately rehydrated, provide consistent analyte recoveries and facilitate accurate control over sample-to-solvent ratios [17]. In this study, 5 mL of water was added to all extraction mixtures to hydrate the freeze-dried samples, demonstrating that the addition of water improves the partitioning efficiency of pesticides during QuEChERS extraction by swelling the matrix and enhancing analyte desorption [20,24]. Similarly, studies on food matrices with a low moisture content showed that insufficient hydration can lead to poor recovery due to the inadequate extraction of target compounds trapped within the matrix [25]. The consistent recovery of most pesticides (70–120%) indicates that the water proportion used was sufficient to optimize extraction without excessively diluting the solvent system. However, slight variations in recovery were observed for some lipophilic pesticides, which could be influenced by the balance between solvent volume and water content. Excessive water may reduce the extraction efficiency of non-polar pesticides by increasing the polarity of the extraction environment, thus hindering their transfer into the organic phase.

The quality of the extract was also enhanced by the addition of MgSO_4_ and Na_3_ Citrate, which facilitated phase separation and effectively removed residual water. This improvement is essential when dealing with insect matrices that are high in fat and susceptible to emulsification. These findings are consistent with recent food safety studies that employed QuEChERS-based extraction protocols [10,26].

### 2.2. Optimization of MRM Condition for GC–MS/MS Analysis

To optimize pesticide detection, collision energy (CE) tests were conducted to identify the two most suitable ion transitions—primary and secondary—from precursor to product ions for multiple reaction monitoring (MRM). Quantification was performed by GC-MS/MS using the external standard method. The Agilent Mass Hunter software (version 10.2) was used to determine the peak area of each analyte’s primary ion transition.

**Figure 2:**
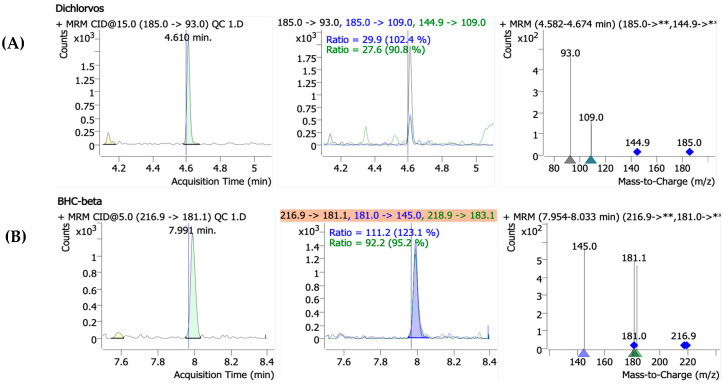
The representative mass spectra of the selected pesticide standards obtained under collision-induced dissociation (CID) conditions. (**A**) Dichlorvos** The protonated molecular ion [M+H]^+^ at *m*/*z* 185.0 yields fragment ions at *m*/*z* 93.0 and 109.0. (**B**) BHC-beta** The precursor ion [M+H]^+^ at *m*/*z* 216.9 generates a fragment ion at *m*/*z* 181.1, while another ion at *m*/*z* 218.9 fragments into *m*/*z* 183.1.

Precursor ions were identified through full-scan analysis for each pesticide, and their retention times (RTs) were determined under the specified gas chromatography (GC) conditions. Product ion scans were then carried out at varying collision energies (0–50 eV) using the selected precursor ions. The optimal MRM conditions were established by evaluating the sensitivity of the resulting product ions at varying CE levels. For confirmation, two product ions per precursor, along with their ion ratios and retention times, were selected. The qualitative and quantitative ions were selected based on their signal intensity and minimal interference from surrounding ions or baseline noise, as detailed in Table 1, and Figure 2 shows the mass spectra of the representative pesticide standards obtained under collision-induced dissociation (CID) conditions, including the ion ratios for dichlorvos and β-BHC.

### 2.3. Method Validations

#### 2.3.1. Linearity, Selectivity, Limit of Detection, and Limit of Quantification

The selectivity of the analytical method was evaluated by comparing chromatograms of the recovery samples with those of standard solutions and blank samples. All 47 pesticide compounds demonstrated consistent retention times and m/z values under the established method using matrix-matched standards, with no interfering peaks observed. These results confirm the high selectivity and effective separation achieved by the developed analytical technique. The limits of detection (LODs) for individual pesticides ranged from 1 to 5 μg/kg, while the limits of quantification (LOQs) ranged from 10 to 15 μg/kg. Linearity, which refers to the method’s ability to produce results directly proportional to the analyte concentration within a specified range, was assessed using matrix-matched calibration curves. The GC–MS/MS results, summarized in Table 2 and Table 3, include correlation coefficients (R^2^), y-intercepts, and calibration curve parameters. The R^2^ values ranged from 0.9940 to 0.9999, and the linearity of the method was evaluated by assessing the percentage deviation of the back-calculated concentration (BBC) from the nominal concentration at each calibration level. The % deviations of the BBC at each calibration level were calculated and reported following the criteria specified in Table 2, which were used according to the SANTE guidelines [24]. The results demonstrated that the majority of the pesticide compounds (47 analytes) had acceptable limits across all levels, indicating excellent linearity. Most compounds exhibited deviation values below ±30%, while, at mid- to high levels (10–500 µg/kg), deviations generally fell within ±20%. These findings confirm that the analytical method demonstrates excellent linearity for quantitative analysis.

#### 2.3.2. Precision and Accuracy

The accuracy and precision of the target compounds in the developed method were assessed based on average recovery and relative standard deviation (RSD) from five trials (*n* = 5). Three spiking concentrations—low, medium, and high—were selected based on the linear range of the target compounds. For a linear range of 5 to 500 μg/kg, the spiking levels were set at 10 μg/kg (low), 100 μg/kg (medium), and 500 μg/kg (high). Recovery rates ranged from 70% to 120%, with RSD values below 20%, indicating the method’s effectiveness. In the recovery test using GC–MS/MS for edible insects, all pesticides exhibited recovery within the acceptable range of 70–120%. Additionally, RSD values were confirmed to be under 20% for all pesticides, as shown in Table 3.

In our study, acetonitrile was selected as the extraction solvent for the multiresidue analysis of pesticides in edible insect samples. In addition, to achieve better efficiency in the extraction and recovery of multiresidue pesticides from the samples, the QuEChERS procedure was employed in our study, as all recovery rates fell within the range considered effective and promising. The QuEChERS procedure yielded extraction recoveries ranging from 64.54% to 122.12%, with RSDs between 1.86% and 6.02%. On the other hand, the QuEChERS procedure may have caused strong salting-out effects, as the mean recoveries of methamidophos (122.12%) treated at a level of 100 μg/kg were observed to be higher than those of other pesticides [19,27]. However, such salting-out effects were not observed in our study. Therefore, the recoveries of multiresidue pesticides were generally between 70 and 120% with RSDs below 20%, indicating that the proposed method in this study was feasible to analyze 47 pesticides in samples.

The validated method developed in this study demonstrates performance metrics comparable to and, in some aspects, exceeding those reported in the recent literature. The technique achieved strong linearity (R^2^ = 0.9940–0.9999) across all 47 target pesticides, which aligns closely with the other studies in Table 4, and Shin et al. (2020) [19] and Labu et al. (2022) [6], both of which reported R^2^ values > 0.995. The LOQ range (10–15 µg/kg) in this study is slightly higher than the 5 µg/kg commonly reported in several other investigations, such as those by Kim et al. (2020) [17] and Kolakowski et al. (2021) [7], yet remains within acceptable sensitivity limits for regulatory purposes. In terms of recovery, this study showed results from 64.54% to 122.12%, with over 97.87% of the analytes falling within the 70–120% range defined by SANTE. And, relative standard deviations (RSDs) in this study were consistently below 6.02%, indicating excellent repeatability.

**Figure 3:**
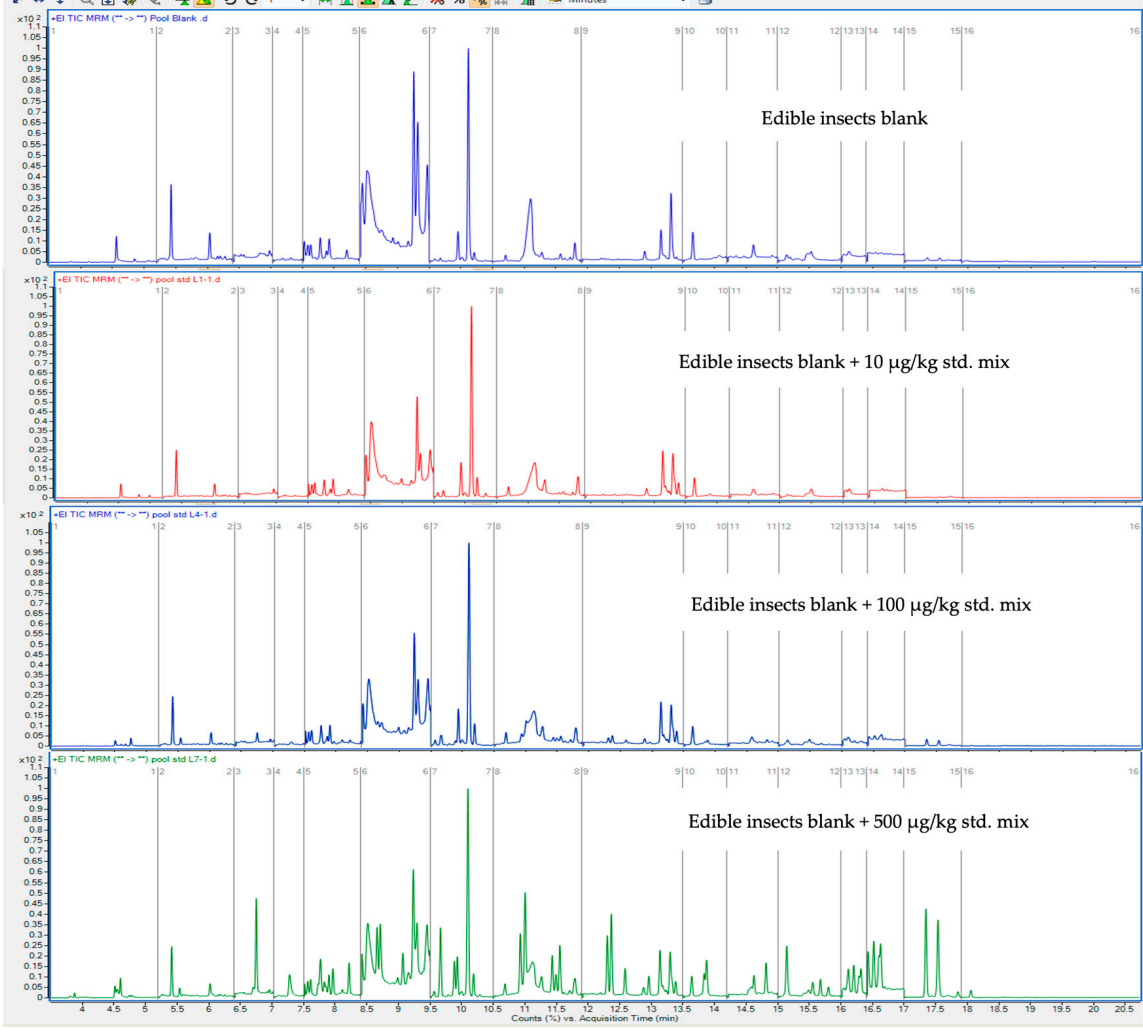
Reconstructed GC-MS/MS chromatograms of edible insects blank and edible insects blank fortified at 10, 100, and 500 μg/kg in 20 min analyses of 47 target compounds with 1 μL injection volume.

Moreover, the relative response of each analyte varied according to its molecular structure and fragmentation pattern. Nevertheless, the sensitivity of the developed GC-MS/MS method was sufficient to screen GC-amenable pesticides at a fortification level of 10 μg/kg using a 2.5 g sample and 15 mL of solvent for extraction. Minimal matrix interference was observed under these conditions, as demonstrated by the comparison of total ion chromatograms between blank edible insect samples and those fortified at 100 and 500 μg/kg (Figure 3). These results confirm the method’s suitability for analyzing trace levels of pesticides in complex, high-fat matrices.

#### 2.3.3. Matrix Effect

The matrix effect provided information about the matrix effects. Depending on the decrease/increase in the percentage of the slope, different matrix effects could be observed: a signal suppression or enhancement effect between −20% and 0% and between 0% and +20% was considered to be mild; a medium effect was supposed when the slope values were between −50% and −20% or +20% and +50%; and a strong effect of signal suppression or enhancement was supposed when values were below −50% or above +50%. These distributions depended not only on the matrix effect but also on the combination of the compound matrix. Pesticides with %ME values within the acceptable range of −20% to +20% experience minimal matrix effects and are less affected by matrix interferences, ensuring more reliable quantification [27,28,29].

Matrix effects (%MEs) observed in the analysis of pesticide residues in edible insect samples varied widely among the compounds, ranging from −33.01% to 24.04%. For instance, fenobucarb and prothiofos exhibited high matrix suppression effects at −33.00% and −33.01%. While omethoate was at +24.24% and endosulfan at +23.70%, respectively, more than 95.74% of the pesticides showed a soft matrix effect (Table 2) with negligible effects in the tested range [29]. Moreover, our findings are in agreement with the study by Łozowicka et al. (2017), who emphasized that modified QuEChERS protocols incorporating buffering salts and increased solvent volumes effectively reduce matrix effects during GC-MS/MS analysis in complex matrices such as soil or insects [10]. However, most of the pesticides in the current investigation were unaffected by the matrix, likely due to the edible insect matrices being effectively eliminated. The extraction procedure effectively removed numerous proteins and lipids that significantly impaired the matrix effect. The dilution procedure used to prepare the sample can also be helpful. Between the extraction and partitioning stages, 5–0 times more solvent was utilized than in traditional QuEChERS procedures [10,30]. Dilution reduced the concentration of the sample matrices to a point where the signal remained unaffected. However, using this method, a small percentage of pesticides (4.25%) exhibited a moderate matrix effect (Table 3). Therefore, for accurate quantification, a matrix-matched calibration procedure ought to be employed.

**Table 4:** Previous studies’ analytical methods for determining pesticide residues in edible insects.

Matrix	Instrument	No. Analysis	RSD (%)	Recovery (%)	LOQ (µg/kg)	Linearity (*r*^2^)	Matrix Effect (ME %)	Reference
Mealworms	LC-MS/MS	353	5–15	75–115	5	>0.995	−20 to +25	[19]
Mealworms	GC-MS/MS, LC-MS/MS	300	5–12	80–115	5	>0.993	−15 to +20	[17]
Edible insects	GC-MS/MS, LC-MS/MS	-	5–20	75–120	5	>0.990	−18 to +22	[7]
Edible insects (6 species)	LC-MS/MS	374	6–15	75–110	5	>0.995	−15 to +25	[6]
Mealworm larvae	GC-MS/MS	247	0–19.9	70–120	50	≥0.990	Not specified	[31]

## 3. Materials and Methods

### 3.1. Chemicals and Materials

Standard pesticide mixtures with a purity of at least 99% were obtained from Dr. Ehrenstorfer (Augsburg, Germany). The combinations comprised a total of 47 chemicals. A diverse array of chemical classes was incorporated—organophosphates (OPs), carbamates (CBs), pyrethroids (PYs), and organochlorines (OCs). Pesticides commonly used in agricultural activities particularly relevant to crops and associated with edible insect habitats were prepared at a concentration of 1000 µg/mL in acetonitrile. For standard fortification, a composite pesticide stock solution was established with various concentrations of standard curve pesticides ranging from 5 to 500 μg/L. A standard mix was used to fortify 2.5 g of pooled edible insect blank by adding 100 μL of the mix to the sample prior to extraction. HPLC-grade acetonitrile, ethyl acetate, and water were obtained from J.T. Baker (Avantor, Radnor Township, PA, USA). They were used for GC/MS and solvent extraction. QuEChERS original packet 50 mL centrifuge tubes filled with 4 g of MgSO_4_, 1 g of NaCl, 1 g of Na_3_C_6_H_5_O_7_·2H_2_O, and 0.5 g of Na_2_HC_6_H_5_O_7_·1.5H_2_O from Agilent Technologies, Santa Clara, CA, USA. Additionally, dispersive cleanup tubes (2 mL) were sourced, containing 150 mg of MgSO_4_, 50 mg of PSA sorbent, bulk carbograph, and 50 mg of end-capped C-18 sorbent, also from Agilent Technologies, USA. For GC-MS/MS, helium (99.999%) and nitrogen (99.999%) served as the carrier gas and collision gas, respectively.

### 3.2. Sample Selection

In June and July 2023, edible insects were gathered from a local market in Chiang Mai, including bamboo caterpillars, house crickets, silkworm pupae, giant water bugs (*Lethocerus indicus*), and grasshoppers. A total of 200 g of edible insect samples were collected and freeze-dried at −50 °C for at least 72 h using an SP VirTis Genesis Pilot Freeze Dryer and the Genesis 25 L Pilot Lyophilizer (Westminster, CO, USA). After freeze-drying, the samples were ground into a fine powder. A sample blank was produced by combining multiple individual insect specimens into a single, homogenized unit in studies that involve consumable insects. The non-fortified sample was also prepared to create a blank matrix for matrix-matched standards. Each sample, weighing 2.5 g, was placed in a 50 mL centrifuge tube (Corning Inc., Wujiang, China) and stored at −20 °C until analysis.

### 3.3. Sample Extraction

Pesticide residues in the edible insect samples were extracted using the EN QuEChERS method [26], with slight modifications. Prior to extraction, the samples were brought to room temperature and spiked with an appropriate standard mixture to achieve final concentrations of 10, 100, and 500 μg/kg. A non-fortified (blank) sample was also prepared to serve as a blank matrix for matrix-matched standards. For the extraction, 5 mL of purified water and 15 mL of acetonitrile were added to each sample tube and mixed thoroughly. The tubes were then tightly capped and shaken for 5 min using a SPEX 2000 Geno Grinder (Digital Multi-Tube Vortex Mixer, Model VXMTDG, OHAUS, Parsippany, Morris County, NJ, USA) at 2500 rpm. Following this, a QuEChERS extraction packet containing 4 g of MgSO_4_, 1 g of NaCl, 1 g of Na_3_C_6_H_5_O_7_·2H_2_O, and 0.5 g of Na_2_HC_6_H_5_O_7_·1.5H_2_O was added. The contents were shaken again for 5 min at the same speed and then centrifuged at 2000 rpm for 5 min.

Next, approximately 1 mL of the upper (organic) layer of the extract was transferred into a 2 mL dispersive solid-phase extraction (SPE) tube containing 150 mg of MgSO_4_, 50 mg of PSA sorbent, bulk carbograph, and 50 mg of end-capped C18 sorbent. The tube was capped, vortexed for 1 min, and centrifuged at 1300 rpm for 3 min. The resulting upper layer was filtered through a 0.45 µm syringe filter and transferred to sample vials for analysis. Finally, 1 μL was injected into a Gas Chromatography Triple Quadrupole Mass Spectrometer (GC-MS/MS) using an autosampler.

### 3.4. GC-MS/MS Instrument Conditions

GC-MS/MS analysis was performed using an Agilent 8890 Gas Chromatograph, equipped with a 7693A autosampler and a 7000E triple quadrupole mass spectrometer, operated through Mass Hunter Software (Qualitative Analysis version B.12.0.430.0 and Quantitative Analysis version B.12.1.938.3) installed on a dedicated workstation for data acquisition and processing (Agilent Technologies, Inc., Santa Clara, CA, USA). The GC and backflush method parameters, including the tandem mass spectrometry and MRM (multiple reaction monitoring) mode settings, are detailed in Table 5 and Table 6. Analyte separation was achieved using two HP-5 ms Ultra Inert capillary columns from Agilent (0.25 mm internal diameter × 30 m length, 0.25 μm film thickness), connected via a backflush union.

### 3.5. Method Validation

#### 3.5.1. Linearity and Sensitivity

The linearity of all compounds in both the solvent and mixed standard solutions was evaluated by plotting the peak area against standard concentrations of 5, 10, 25, 50, 100, 250, and 500 µg/kg. Calibration curves were constructed using a 1/x weighting factor, excluding the origin. The coefficient of determination (R^2^) was calculated to assess the accuracy of the calibration model, with an acceptance criterion of R^2^ ≥ 0.9900. Moreover, the linearity of the method was evaluated by assessing the percentage deviation of the back-calculated concentration (BBC) from the nominal concentration at each calibration level. According to the criteria, the deviation should not exceed ±20% at all concentration levels. Instrument sensitivity was assessed by determining the limit of detection (LOD) and the limit of quantification (LOQ). The LOD was defined as the lowest concentration at which a pesticide signal could be reliably distinguished from the background noise at the corresponding retention time. Following the SANTE/11312/2021 guidelines [24], signal-to-noise (S/N) ratios of 3 and 10 were used to define the LOD and LOQ, respectively.

#### 3.5.2. Precision and Accuracy

The accuracy of the method was evaluated through recovery studies, while precision was assessed based on the relative standard deviation (RSD) [24]. For this purpose, a mixture of standard solutions was spiked into a pooled blank sample of edible insects. Recovery and precision were determined at three fortification levels—10, 100, and 500 µg/kg (*n* = 3 for each level)—using matrix-matched standards. The precision of the method was further confirmed by calculating RSD of the analyte responses across replicates.

#### 3.5.3. Matrix Effect

The matrix effect (ME) of each analyte was assessed using a mass spectrometry-based analytical method [24]. To determine the ME of the pesticides in each sample, the linear regression slope of the matrix-matched calibration curve was compared to the linear regression slope of the standard solution calibration without the matrix using the following Equation (1).

(1) $$\mathrm{Matrix}\;\mathrm{effect}\;\%=\left[\frac{\mathrm{Slope}\;\mathrm{of}\;\mathrm{matrix}\;\mathrm{matched}\;\mathrm{calibration}\;\mathrm{curve}}{\mathrm{Slope}\;\mathrm{of}\;\mathrm{standard}\;\mathrm{solution}\;\mathrm{calibration}\;\mathrm{curve}}\right]-1\times 100$$

## 4. Conclusions

A thorough multiresidue analytical technique was devised and validated using GC-MS/MS for the simultaneous identification of 47 pesticides in high-fat edible insect matrices. The refined QuEChERS extraction technique, utilizing acetonitrile and dispersive solid-phase extraction with MgSO_4_, PSA, and C18, facilitated the efficient elimination of lipids and moisture while maintaining analyte integrity. Method validation was performed in accordance with SANTE guidelines. Most pesticides exhibited adequate recovery rates (70–120%) and relative standard deviations of less than 5% overall. Despite several chemicals demonstrating reduced recovery at the lowest concentration level, the overall technique performance stayed within acceptable regulatory parameters. Matrix-matched calibration proficiently mitigated matrix effects, guaranteeing precise quantification. This established technology is suitable for regular pesticide monitoring in consumable insects, supporting food safety oversight and regulatory compliance.

## Figures and Tables

**Table 1:** The MRM Conditions of the primary and secondary transitions of a precursor to product ions for pesticides.

No.	Pesticides	Groups	RT (min)	Precursor > Product Ion (CE, eV)		Dwell
				Quantifier Ion	Qualifier Ion	
1	Methamidophos	OP	4.581	141 > 95 (5 eV)	141> 80 (20 eV)	10
2	Dichlorvos	OP	4.610	184.9 > 93 (10 eV)	144.9 > 109 (10 eV)	10
3	Mevinphos	OP	5.557	127 > 10 (10 eV)	192> 126 (10 eV)	10
4	Acephate	OP	5.663	136 > 94 (15 eV)	78.9 > 47 (10 eV)	10
5	Omethoate	OP	6.850	110 > 79 (15 eV)	156 > 79 (25 eV)	10
6	Fenobucarb	CB	6.769	121 > 77 (20 eV)	149.9 > 121.1 (5 eV)	10
7	Monocrotophos	OP	7.380	127.1 > 109 (10 eV)	127.1 > 95 (15 eV)	10
8	Dimethoate	OP	7.806	87 > 46 (20 eV)	125 > 47 (15 eV)	10
9	Carbofuran	CB	7.781	164.2 > 149.1 (10 eV)	149.1 > 121.1 (5 eV)	10
10	Atrazine	Herbicide	7.847	214.9 > 58.1 (10 eV)	200 > 122.1 (5 eV)	10
11	BHC-beta	OC	7.991	216.9 > 181.1 (5 eV)	218.9 > 183.1 (5 eV)	10
12	Diazinon	OP	8.239	137.1 > 84 (10 eV)	199.1 > 93 (15 eV)	10
13	Pirimicarb	CB	8.685	238 > 166.2 (10 eV)	166 > 55.1 (20 eV)	10
14	Parathion-methyl	OP	9.091	262.9 > 109 (10 eV)	125 > 47 (10 eV)	10
15	Fenitrothion	OP	9.094	125.1 > 47 (15 eV)	277 > 109 (5 eV)	10
16	Pirimiphos-methyl	OP	9.096	232.9 > 151 (5 eV)	232.9 > 125 (5 eV)	10
17	Carbaryl	CM	9.250	144.1 > 116.1 (15 eV)	144.1 > 89 (45 eV)	10
18	Heptachlor	OC	9.279	271.7 > 236.9 (15 eV)	273.7 > 238.9 (15 eV)	10
19	Malathion	OP	9.672	126.9 > 99 (5 eV)	172.9 > 99 (15 eV)	10
20	Aldrin	OC	9.880	262.9 > 192.9 (35 eV)	263 > 228 (20 eV)	10
21	Chlorpyrifos	OP	9.904	196.9 > 169 (15 eV)	198.9 > 171 (15 eV)	10
22	Methidathion	OP	10.952	144.9 > 85 (5 eV)	144.9 > 58.1 (15 eV)	10
23	DDE-o,p′	OC	11.024	246 > 176.2 (30 eV)	248 > 176.2 (30 eV)	10
24	Endosulfan	OC	11.197	158.9 > 89 (35 eV)	194.9 > 124.9 (30 eV)	10
25	Prothiofos	OP	11.447	113 > 94.9 (10 eV)	266.9 > 239 (5 eV)	10
26	Profenofos	OP	11.500	207.9 > 63 (30 eV)	338.8 > 268.7 (15 eV)	10
27	DDE-p,p′	OC	11.569	246.1 > 176.2 (30 eV)	246 > 211 (15 eV)	10
28	DDT-o,p′	OC	12.382	235 > 165.2 (20 eV)	235 > 199.1 (15 eV)	10
29	Ethion	OP	12.382	231 > 129 (10 eV)	231 > 175 (5 eV)	10
30	Triazophos	OP	12.609	161.2 > 134.2 (5 eV)	161.2 > 91 (15 eV)	10
31	Triphenyphosphate	OP	13.315	214.9 > 168.1 (15 eV)	232.9 > 215.1 (10 eV)	10
32	Carbosulfan	CM	13.778	118 > 76 (5 eV)	164 > 103.1 (25 eV)	10
33	EPN	OP	13.897	169 > 141.1 (5 eV)	169 > 77.1 (25 eV)	10
34	Phosmet	OP	13.959	160 > 77.1 (20 eV)	160 > 133.1 (10 eV)	10
35	Amitraz	Acaricide/Insecticide	14.714	132.1 > 117.1 (15 eV)	162 > 132.2 (5 eV)	10
36	Azinphos-methyl	OP	14.610	160 > 77 (20 eV)	132.1 > 77 (15 eV)	10
37	Cyhalothrin (Lambda)	PY	14.834	208.1 > 181.1 (10 eV)	181.1 > 152.1 (30 eV)	10
38	Fenpropathrin	PY	14.834	181.1 > 152.1 (25 eV)	181 > 127 (5 eV)	10
39	Azinphos-ethyl	OP	15.238	132 > 77.1 (15 eV)	160 > 77.1 (20 eV)	10
40	Permethrin	PY	15.688	183.1 > 168.1 (10 eV)	183.1 > 153.1 (15 eV)	10
41	Coumaphos	PY	15.844	210 > 182 (10 eV)	361.9 > 109 (15 eV)	10
42	Cyfluthrin I	PY	16.172	162.9 > 90.9 (15 eV)	226 > 206 (5 eV)	10
43	Cyfluthrin II	PY	16.253	162.9 > 90.9 (15 eV)	162.9 > 127 (5 eV)	10
44	Cyfluthrin III	PY	16.254	162.9 > 90.9 (15 eV)	162.9 > 127 (5 eV)	10
45	Cyfluthrin IV	PY	16.367	162.9 > 90.9 (15 eV)	162.9 > 127 (5 eV)	10
46	Cypermethrin I	PY	16.454	163 > 91 (10 eV)	226 > 206 (5 eV)	10
47	Cypermethrin II	PY	16.543	163.1 > 127.1 (5 eV)	163.1 > 91 (15 eV)	10
48	Cypermethrin III	PY	16.628	163.1 > 127.1 (5 eV)	163.1 > 91 (15 eV)	10
49	Cypermethrin IV	PY	16.651	163.1 > 127.1 (5 eV)	163.1 > 91 (15 eV)	10
50	Fenvalerate	PY	17.561	167 > 125.1 (5 eV)	208.9 > 141.1 (15 eV)	10
51	Fluvalinate-tau	PY	17.558	250 > 55 (40 eV)	181 > 152 (40 eV)	10
52	Esfenvalerate	PY	17.560	167 > 125.1 (10 eV)	181 > 152.1 (25 eV)	10
53	Deltamethrin	PY	18.082	252.9 > 93 (15 eV)	181 > 152.1 (25 eV)	10

RT = retention time, CE = collision energy, eV = electron volt. Pesticide groups: organophosphates (OPs), carbamates (CBs), pyrethroids (PYs), organochlorines (OCs).

**Table 2:** The linearity of the analytical method based on the % deviation of back-calculated concentrations (BBCs) in edible insect samples (*n* = 5).

No.	Pesticides	Calibration Levels (µg/kg)	% Deviation of BBC						
			5 µg/kg	10 µg/kg	25 µg/kg	50 µg/kg	100 µg/kg	250 µg/kg	500 µg/kg
1	Methamidophos	5–500	15.00	5.90	2.12	−15.87	−13.28	4.23	−0.45
2	Dichlorvos	5–500	7.60	11.11	−1.91	−3.21	−3.61	−4.44	1.26
3	Acephate	5–500	−13.20	16.59	−5.28	5.86	−0.79	−12.56	0.49
4	Mevinphos	5–500	4.00	2.30	−6.80	−2.06	−6.15	−2.95	−3.15
5	Fenobucarb	5–500	8.60	1.65	11.90	−8.64	−3.81	2.94	1.44
6	Omethoate	5–500	10.80	12.00	−14.03	−11.17	−5.59	−1.67	0.47
7	Monocrotophos	5–500	5.40	10.88	−13.48	−3.93	−8.83	4.23	0.50
8	Dicrotofos	5–500	−14.60	18.62	−13.35	4.09	−8.22	−4.05	0.51
9	Carbofuran	5–500	14.91	−8.25	−0.65	8.79	8.27	−1.18	−0.09
10	Dimethoate	5–500	8.60	−5.59	−22.40	−1.31	2.02	−1.13	0.40
11	Atrazine	5–500	−2.27	17.08	4.72	−4.64	−2.36	1.03	−0.14
12	BHC-beta	5–500	−15.20	−26.36	9.23	−9.91	−15.13	−1.19	0.93
13	Diazinon	5–500	3.20	−15.44	−8.74	−1.99	4.02	4.06	−1.12
14	Pirimicarb	5–500	14.14	−4.21	−6.57	−2.36	2.40	3.50	−0.92
15	Parathion-methyl	5–500	4.07	−6.45	−10.91	2.94	1.70	5.34	0.34
16	Fenitrothion	5–500	4.40	−9.73	−18.16	−11.32	4.45	5.69	0.31
17	Pirimiphos-methyl	5–500	−1.20	−9.95	9.16	−10.72	9.20	−4.14	0.42
18	Carbaryl	5–500	13.60	−15.57	−13.24	−12.33	−5.16	−0.59	0.50
19	Heptachlor	5–500	−13.00	−16.18	1.72	−4.09	−7.12	−0.28	2.34
20	Malathion	5–500	−11.20	0.69	−8.04	−11.93	−6.05	−1.79	0.29
21	Aldrin	5–500	5.00	1.35	−11.80	−1.96	6.12	6.94	−1.91
22	Chlorpyrifos	5–500	9.48	11.20	1.05	−7.10	−6.10	0.28	0.22
23	Methidathion	5–500	4.40	16.46	−13.08	−4.76	7.63	−1.86	0.41
24	DDE-o,p′	5–500	2.40	6.28	−11.70	0.48	8.06	6.38	−1.86
25	Endosulfan	5–500	15.60	3.30	7.55	16.86	18.97	14.97	−0.86
26	Prothiofos	5–500	0.60	17.41	4.05	−10.45	−6.54	−0.09	0.35
27	Profenofos	5–500	−0.40	13.88	11.71	−16.18	4.99	−15.45	1.06
28	DDE-p,p′	5–500	−17.80	−9.33	−13.26	−0.98	5.47	6.24	−1.71
29	DDT-o,p′	5–500	−17.80	−19.65	18.98	0.70	−16.90	−5.07	1.84
30	Ethion	5–500	15.20	19.46	18.70	−13.01	−0.40	−6.08	2.34
31	Triazophos	5–500	12.22	−19.42	−9.26	−0.41	−7.67	−5.62	0.37
32	Cabosulfan	5–500	2.21	−6.79	15.80	−11.43	−2.20	−17.52	0.91
33	EPN	5–500	−11.44	3.45	−14.72	−11.45	−1.77	−7.85	2.81
34	Phosmet	5–500	−20.90	12.31	11.61	5.14	−5.51	7.81	0.47
35	Amitraz	5–500	15.93	1.58	1.09	−4.15	−6.42	−0.08	0.29
36	Azinphos-methyl	5–500	−2.00	19.70	−16.77	−7.35	−14.98	−2.13	−2.76
37	Cyhalothrin (Lambda)	5–500	4.22	1.87	18.73	−10.88	−16.34	−5.26	1.97
38	Fenpropatrin	5–500	−12.39	−0.15	17.54	−10.73	−17.61	−3.45	1.57
39	Azinphos-ethyl	5–500	1.39	−12.96	−0.72	−9.53	−3.62	0.66	0.43
40	Permethrin	5–500	−11.92	10.89	−0.34	−5.97	−4.53	2.37	−0.36
41	Coumaphos	5–500	7.29	6.81	−12.20	5.00	3.23	−1.04	0.50
42	Cyfluthrin	5–500	−13.02	0.17	5.09	−6.12	−17.69	−0.59	0.69
43	Cypermethrin	5–500	−6.64	5.29	3.66	−3.77	3.16	−6.58	1.64
44	Fenvalerate	5–500	2.93	1.41	8.03	−14.12	3.37	−6.92	1.81
45	Esfenvalerate	5–500	12.69	5.08	−3.55	−15.66	−14.34	−5.73	2.05
46	Fluvalinate-tau	5–500	2.55	14.84	4.57	−11.90	0.08	3.79	−0.67
47	Deltamethrin	5–500	−14.03	5.22	−6.69	−17.73	−11.12	−1.41	0.87

BBC = back-calculated concentration. % deviation of the back-calculated concentration from the true concentration ≤ ±20.

**Table 3:** Limit of detection (LOD), limit of quantification (LOQ), recovery, precision, and matrix effect in pooled edible insect samples by GC-MS/MS (*n* = 5).

No.	Pesticides	Linearity (*r*^2^)	Limit of Detection Limit (LOD)	Limit of Quantification (LOQ)	Low (10 μg/kg) Spike Level		Medium (100 μg/kg) Spike Level		High (500 μg/kg) Spike Level		ME %
			μg/kg	μg/kg	Recovery (%)	RSD (%)	Recovery (%)	RSD (%)	Recovery (%)	RSD (%)	
1	Methamidophos	0.9979	1	10	86.72	3.77	122.12	1.96	99.55	2.81	6.13
2	Dichlorvos	0.9989	1	10	96.39	2.71	98.09	2.15	101.26	2.24	−1.60
3	Acephate	0.9939	2	10	64.54	4.77	94.72	3.32	100.49	3.40	14.33
4	Mevinphos	0.9948	1	10	100.37	2.57	87.57	2.39	98.53	2.23	−15.40
5	Fenobucarb	0.9986	2	10	96.19	2.63	111.90	2.08	101.44	2.25	−33.00
6	Omethoate	0.9936	3	15	88.50	4.42	85.97	3.72	100.47	2.99	24.24
7	Monocrotophos	0.9938	2	10	80.12	3.85	86.52	4.62	100.5	2.98	−9.59
8	Dicrotofos	0.9934	5	10	87.40	2.50	86.65	3.14	100.51	3.04	3.13
9	Carbofuran	0.9993	1	10	108.27	3.82	99.35	2.61	99.91	2.38	−11.80
10	Dimethoate	0.9958	1	10	70.38	4.33	82.73	2.48	100.4	2.39	−2.18
11	Atrazine	0.9999	1	10	97.64	3.18	104.72	1.86	99.86	2.29	−13.40
12	BHC-beta	0.9979	1	10	84.87	2.38	109.23	2.06	100.93	2.24	−4.21
13	Diazinon	0.9991	2	10	104.02	2.47	91.26	1.90	98.88	2.16	9.90
14	Pirimicarb	0.9994	1	10	102.40	2.88	93.43	2.28	99.08	2.22	0.22
15	Parathion-methyl	0.9970	1	10	86.40	2.57	89.09	2.32	100.34	2.89	−17.40
16	Fenitrothion	0.9975	3	10	85.00	3.23	81.84	2.43	100.31	3.07	6.06
17	Pirimiphos-methyl	0.9953	1	10	79.40	4.52	109.16	3.18	100.42	2.74	−10.08
18	Carbaryl	0.9969	1	10	94.84	2.83	86.76	2.03	100.5	2.13	8.68
19	Heptachlor	0.9961	1	10	92.88	4.49	101.72	4.64	102.34	3.10	4.74
20	Malathion	0.9978	1	10	100.00	2.45	91.96	2.12	100.29	2.25	4.63
21	Aldrin	0.9975	3	10	106.12	4.16	88.20	2.04	98.09	2.14	−3.57
22	Chlorpyrifos	0.9996	3	10	93.90	2.69	101.05	1.96	100.22	2.21	−5.01
23	Methidathion	0.9957	1	10	72.25	2.54	86.92	2.37	100.41	2.36	−4.82
24	DDE-o,p′	0.9975	1	10	108.06	3.11	88.30	1.94	98.14	2.19	6.32
25	Endosulfan	0.9957	3	10	118.97	5.14	107.55	3.48	99.14	4.95	23.70
26	Prothiofos	0.9994	5	10	93.46	2.33	104.05	1.94	100.35	2.17	−33.01
27	Profenofos	0.9944	5	10	74.99	2.89	111.71	2.28	101.06	2.31	1.02
28	DDE-p,p′	0.9980	1	10	105.47	2.72	86.74	2.04	98.29	2.29	−1.30
29	DDT-o,p′	0.9967	1	10	83.10	2.61	118.98	1.97	101.84	2.14	2.16
30	Ethion	0.9941	1	10	79.60	2.33	118.70	2.07	102.34	2.11	10.49
31	Triazophos	0.9967	2	10	83.45	2.49	90.74	2.40	100.37	2.32	3.07
32	Cabosulfan	0.9959	3	10	77.80	2.36	115.80	2.37	100.91	2.48	−2.53
33	EPN	0.9926	1	10	78.23	3.52	98.62	3.74	102.81	4.02	3.45
34	Phosmet	0.9968	3	10	64.54	2.88	111.61	2.43	100.47	2.38	−5.33
35	Amitraz	0.9996	1	10	93.58	2.45	101.09	1.95	100.29	2.17	5.85
36	Azinphos-methyl	0.9909	3	10	85.02	2.83	83.23	2.41	97.24	2.71	19.98
37	Cyhalothrin (Lambda)	0.9960	3	10	83.66	2.67	118.73	1.82	101.97	2.14	−0.88
38	Fenpropatrin	0.9966	3	10	82.39	2.62	117.54	2.03	101.57	2.21	3.62
39	Azinphos-ethyl	0.9954	1	10	80.30	2.82	99.28	2.43	100.43	2.39	9.48
40	Permethrin	0.9996	5	10	95.47	2.87	99.66	2.21	99.64	2.06	14.19
41	Coumaphos	0.9940	1	10	89.40	2.53	87.80	2.50	100.5	2.25	−20.58
42	Cyfluthrin	0.9961	2	10	92.01	3.61	106.86	2.97	101.56	6.02	−0.40
43	Cypermethrin	0.9976	2	10	84.20	4.08	109.24	2.62	101.17	2.45	15.64
44	Fenvalerate	0.9967	1	10	77.04	2.63	109.37	1.91	101.76	2.25	−16.40
45	Esfenvalerate	0.9959	2	10	85.66	2.54	120.45	1.97	102.05	2.33	3.56
46	Fluvalinate-tau	0.9982	3	10	100.08	2.71	104.57	1.86	99.33	2.38	−3.01
47	Deltamethrin	0.9982	5	10	88.88	2.52	98.63	2.11	100.87	3.84	0.48

RSD = relative standard deviation. ME = matrix effect was classified as: suppression (%ME < −20%), enhancement (%ME > +20%), and no effect (%ME between −20% and +20%).

**Table 5:** The GC and backflush method conditions.

Parameter	Condition		
Inlet	MMI Injection mode Spitless		
Carrier gas	Helium		
Inlet flow	1 mL/min		
Inlet temperature	280 °C		
Injection volume	1 µL		
MS transfer line temperature	280 °C		
Oven program	Ramp	Temp	Hold
	Time		
	60 °C	1 min	40 °C/min
	170 °C	0 min	10 °C/min
	310 °C	3 min	
Total Run Time	20.75 min		

**Table 6:** The tandem mass spectrometer and MRM mode condition.

Parameter	Condition
Mode	Electron impact
Transfer line temperature	280 °C
Source temperature	280 °C
Quadrupole temperature	Q1 and Q2 = 150 °C
MS1 resolution	Wind
MS2 resolution	Wind
Collision gas flow	Nitrogen at 1.5 mL/min
Quenching gas flow	Helium at 2.25 mL/min
Detector Gain	10

## Data Availability

The original contributions presented in this study are included in the article. Further inquiries can be directed to the corresponding author.
